# Supplementary material for: Pervasive mRNA uridylation in fission yeast is catalysed by both Cid1 and Cid16 terminal uridyltransferases
Source: PLoS One. 2023 May 23;18(5):e0285576. doi: 10.1371/journal.pone.0285576 (PMC10204976; doi:10.1371/journal.pone.0285576)
Supplement: S1 Fig — (PDF) [file pone.0285576.s001.pdf]

# Figure S1

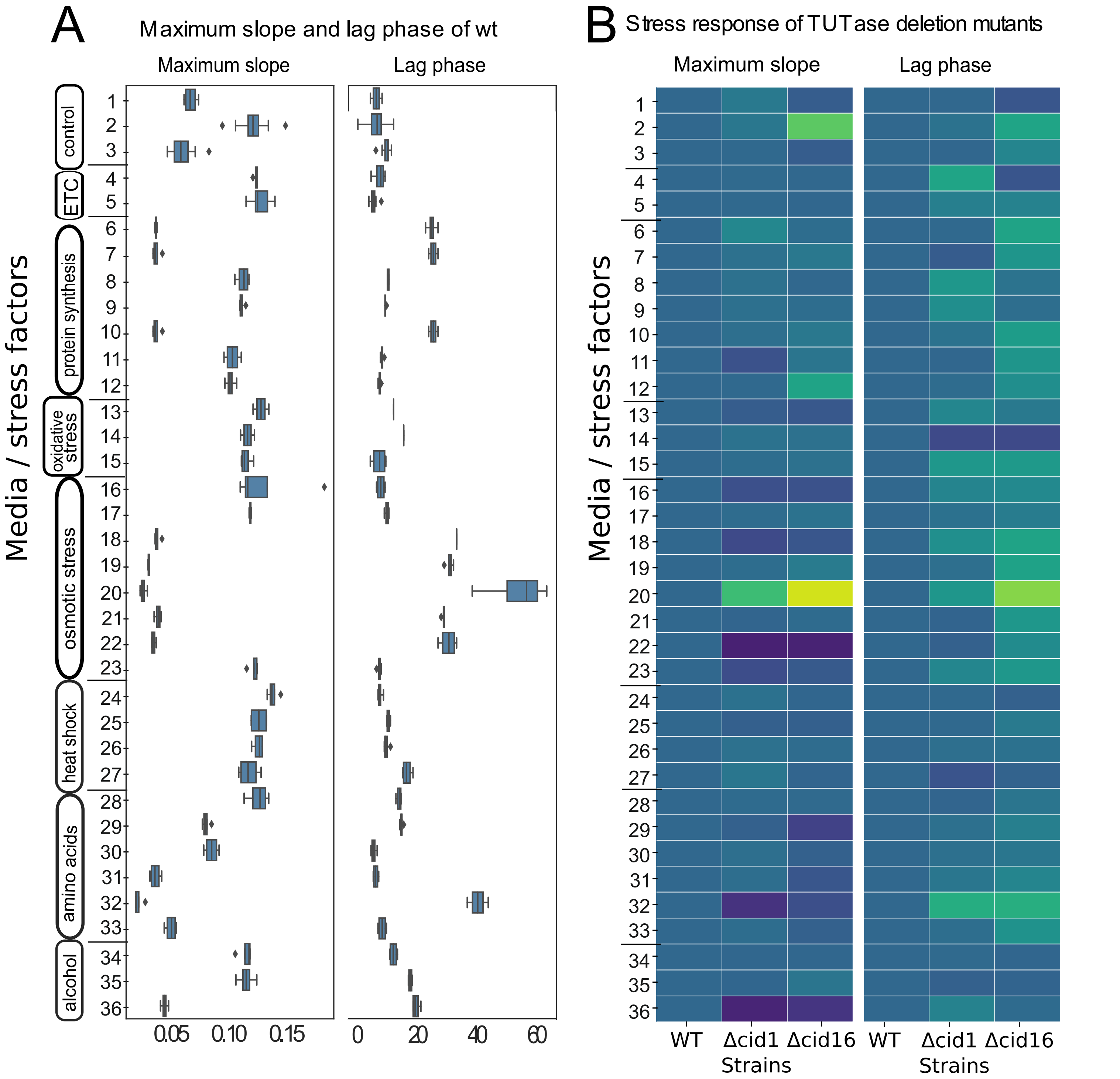


**Fig. S1. Uridylation in fission yeast is dispensable for growth control in normal and stress conditions.** The growth curves for different strains (*WT*, *Δcid1* and *Δcid16*) in different growth conditions were obtained by monitoring changes in optical density (OD 600) using micro-bioreactor Bioscreen C.For each strain data for at least four biological repeats were collected. The maximum slope of the growth curve (describing the growth rate of the culture) and the lag phase (the time required for doubling the initial biomass) were determined using Pyphe growth curves Python module [4].

**A.** Depicts results obtained for WT strain in different media. Maximum slope and lag phase values from different repeats for each condition are visualised in the form of a bar plot.

**B.** Heat map depicting differences between maximum slope and lag phase parameters between wild-type strain and investigated mutants. Averaged results of the maximum slope and lag phase of deletion mutants were normalized to the wild-type strain values for each condition independently. None of the difference detected were statistically significant - statistical analysis was done with one-way ANOVA using Python (SciPy library).
