# Supplementary material for: Pervasive mRNA uridylation in fission yeast is catalysed by both Cid1 and Cid16 terminal uridyltransferases
Source: PLoS One. 2023 May 23;18(5):e0285576. doi: 10.1371/journal.pone.0285576 (PMC10204976; doi:10.1371/journal.pone.0285576)
Supplement: S2 Fig — (PDF) [file pone.0285576.s002.pdf]

Figure S2

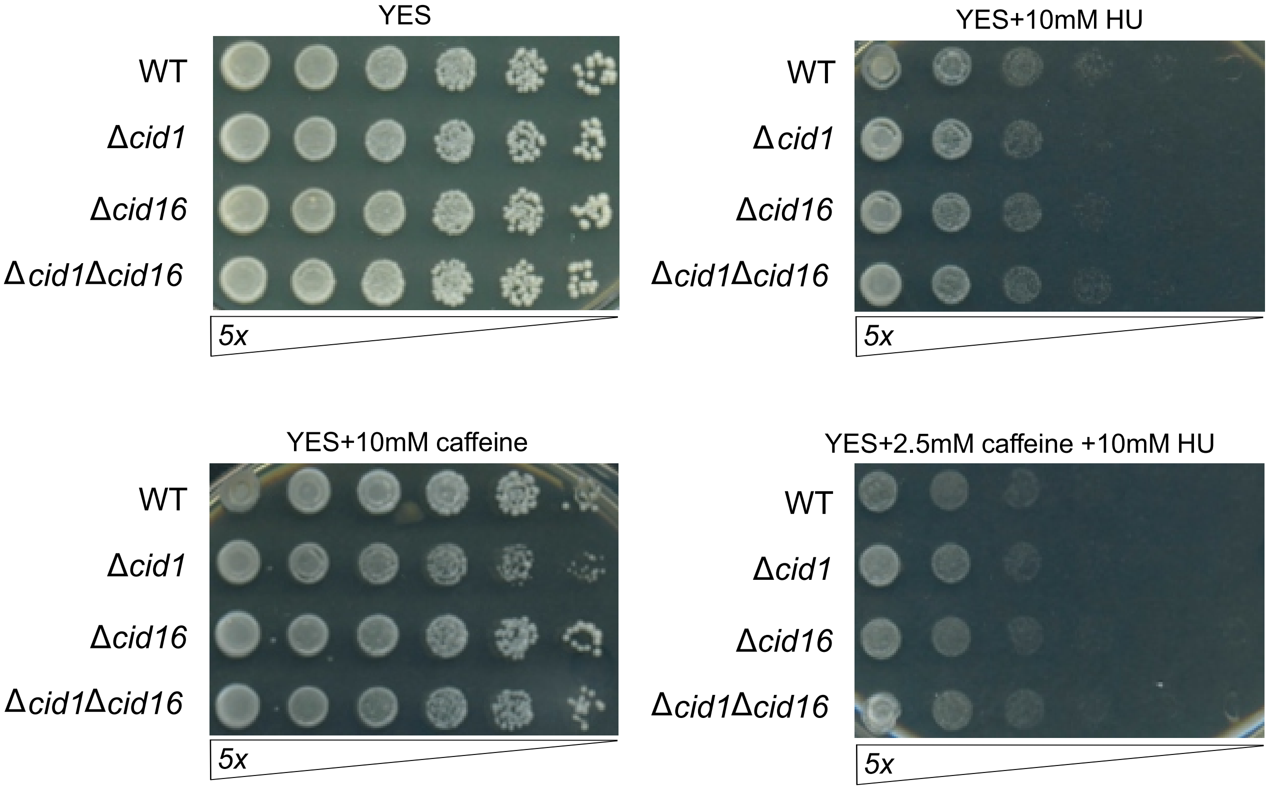

Fig. S2. Terminal uridyltransferases deletion mutants in prototroph background do not exhibit sensitivity to hydroxyurea, caffeine or mix of both.
